# Big data application and firm markups: evidence from China

**DOI:** 10.1038/s41598-026-43480-1

**Published:** 2026-04-08

**Authors:** Dong Wang

**Affiliations:** School of Economics, Jinan University, No. 601 Huangpu Avenue West, Tianhe District, Guangzhou, 510632 Guangdong Province China

**Keywords:** Big data, Markup, Product innovation, Productivity, Environmental social sciences, Mathematics and computing

## Abstract

This study investigates the relationship between big data applications and firms’ price markups. By constructing a heterogeneous firm model with variable markups, we analyze the mechanisms through which big data applications influence firms’ price markups and conduct empirical tests using micro-level firm data. The results indicate that big data applications significantly enhance firms’ price markups. Mechanism analysis reveals that promoting product innovation and improving production efficiency are two key channels through which big data applications contribute to higher markups. Furthermore, the positive effect of big data applications on firms’ markups exhibits heterogeneity across organizational, technological, and environmental dimensions. These findings suggest that while big data applications positively influence firms’ markups, the realization of this effect depends on the synergistic support of various complementary resources. The research uncovers the intrinsic mechanisms through which big data applications shape firms’ competitive advantages and market power, providing valuable insights for policy formulation.

**Supplementary Information:**

The online version contains supplementary material available at 10.1038/s41598-026-43480-1.

## Introduction

In the era of the digital economy, data have become a critical factor of production. A growing number of corporate managers are gradually moving away from decision-making based on intuition and adopting data-driven analytical methods to support more scientific decision-making processes^1^. However, as a byproduct of corporate production activities^2^, data often remain in their raw stored state without effective governance, intelligent analysis, and in-depth mining, making it difficult to transform them into high-value information or structured knowledge that can support decision-making^3^. With the increasing prevalence of general-purpose technologies such as big data, enterprises are now able to integrate and analyze large-scale unstructured data from multiple sources, gaining deeper business insights and thereby building sustainable competitive advantages in the market^4^. Despite the broad application prospects of big data, existing literature primarily focuses on analyzing its impact on short-term productivity and performance^5,6^, while research on how enterprises can derive sustained competitive advantages and market power from such investments remains relatively scarce^7^.

The evolution and evaluation of corporate competitive advantages have always been a focal point in academic research, with indicators such as productivity and sales revenue receiving widespread attention. However, in the context of the digital economy, digital platform enterprises, leveraging the network effects, economies of scale, and flywheel effects of data, are more likely to drive market structures toward monopoly or oligopoly, exhibiting a pronounced “winner-takes-all” characteristic^8^. Moreover, although many enterprises continue to increase their investments in information technology (IT), the full realization of their potential often depends on long-term and systematic investments in intangible assets, including new business processes, business models, and skills training^9^. This makes traditional indicators such as productivity and sales revenue inadequate for timely and comprehensive reflection of the actual transformations, innovations, and indirect benefits occurring within enterprises, leading to the so-called “IT productivity paradox.” In this context, overemphasizing productivity and similar metrics as standards for evaluating corporate competitive advantages may instead trigger more intense price competition, trapping enterprises in an “efficiency paradox” where increased production does not translate into increased profits, while also failing to accurately measure the true benefits brought by IT investments.

In contrast, the firm price markup—the deviation between price and marginal cost—provides a more comprehensive reflection of a firm’s competitive advantage. This indicator not only captures a firm’s performance in both “cost reduction” (lowering marginal costs) and “quality improvement” (raising product pricing) but also offers a more integrated measure of the firm’s ability to transform internal technological investments into sustained competitive advantages^10^. Existing literature has explored the determinants of firm markups from various perspectives, including market competition and industrial concentration^11,12^, international trade^13,14^, and supply and demand shocks^15^. Furthermore, Crouzet & Eberly^16^ and De Ridder^17^ suggest that intangible assets, particularly software-related assets, have a positive impact on markups. This raises an important question: As another form of intangible asset^18^, can big data also enhance firm markups? However, few studies have incorporated big data and firm markups into a unified analytical framework for in-depth investigation.

As a new factor of production in the digital age, data is characterized by non-rivalry, reproducibility, and timeliness^19,20^. Over the past decade, data has experienced explosive growth in scale, variety, and generation speed, often referred to as “big data.” Faced with increasingly vast and complex data resources, how to effectively utilize this data has become a key focus for enterprises. Big data application refers to the process by which enterprises leverage technologies such as data collection, storage, cleaning, analysis, and mining to process massive, multi-source, and rapidly growing data. Through this process, valuable information is extracted and applied to real-world business operations to optimize decision-making and create value^21^. Conceptually, “big data” emphasizes the characteristics of the data itself, while “big data application” focuses more on the practical process of extracting value, supporting decisions, and driving innovation based on data.

Through big data applications, firms can conduct in-depth mining of multi-source, massive, and unstructured data to obtain actionable business insights, thereby driving business transformation and building competitive advantages in the market^22,23^. Existing research has explored the economic consequences of big data adoption by firms, accumulating extensive empirical evidence, particularly regarding firm productivity and performance (e.g^5,6,24–26^). Unfortunately, aside from Eeckhout and Veldkamp^7^, who examined the impact of data on markups from a macroeconomic perspective, research on big data applications and firm markups is nearly absent. Moreover, their study focuses on macroeconomic theoretical reasoning and lacks sufficient micro-level empirical support. Differing from the existing literature, this study provides an in-depth micro-level analysis of the relationship between big data application and firm markups.

Similar to general-purpose technologies such as artificial intelligence, systematic measurement of big data applications at the enterprise level remains relatively scarce, which has become a primary challenge in accurately understanding the economic impact of big data. Existing studies primarily rely on core variable methods and questionnaire surveys to construct measurement indicators for enterprise big data applications. The former often uses metrics such as the number of data analysts as key proxy variables (e.g^3,6,27^). However, big data applications require the synergistic coordination of multiple complementary inputs, making it difficult to capture their full framework using a single indicator alone. The latter mainly depends on structured survey tools (e.g^21,28^), which still struggle to completely avoid subjective biases at the methodological level. Corporate annual reports, with their broad coverage of listed companies, high authority, and comprehensive content, serve as an ideal data source for constructing big data application metrics. However, these reports often contain substantial noise^29^, making effective identification and processing of noise crucial for improving measurement accuracy. Large language models (LLMs), as cutting-edge tools in artificial intelligence, demonstrate significant potential in identifying textual noise and extracting unstructured information. For instance, Li et al.^30^ utilized LLMs to measure corporate culture and its economic consequences from listed companies’ annual reports. Fang et al.^31^ extracted unstructured information, such as policy objectives, target industries, policy tools, and implementation mechanisms, from 3 million Chinese industrial policy texts. This study innovatively employs LLMs to deeply mine corporate annual report texts, aiming to construct a more objective and precise measurement system for enterprise big data applications.

Based on this, this paper examines the impact of enterprise big data applications on price markups and their underlying mechanisms, using Chinese A-share listed companies from 2002 to 2023 as the research sample. Compared to existing studies, the contributions of this paper are mainly reflected in the following three aspects: First, from a micro-enterprise perspective, it integrates big data applications and enterprise price markups into a unified analytical framework, systematically investigating the impact of big data applications on price markups. This extends the research of Eeckhout and Veldkamp^7^ while broadening the theoretical boundaries of big data’s influence in the microeconomic domain. Second, it adopts an innovative measurement method based on large language models to scientifically and accurately gauge the level of big data application at the enterprise level, significantly enhancing the objectivity and precision of indicator construction. This provides a reliable data foundation for empirical analysis and offers a referential measurement tool for subsequent identification of big data applications at the micro level. Third, building on the theoretical analytical framework of Antoniades^32^, it constructs a heterogeneous firm variable markup model to theoretically elucidate how big data applications positively influence enterprise price markups through two key mechanisms: promoting product innovation and enhancing production efficiency. This further deepens the understanding of the mechanisms through which data empowers competitive advantages for enterprises.

The structure of the remaining parts of this paper is as follows: Section "Analytical Framework and Theoretical Model" presents the analytical framework and theoretical model; Section "Research Design" outlines the research design; Section "Empirical Results and Analysis" displays the regression results; Section "Mechanism Analysis" conducts mechanism tests; Section "Heterogeneity Analysis" performs heterogeneity analysis; and the final section concludes with implications.

## Analytical framework and theoretical model

### Analytical framework

**Fig. 1:**
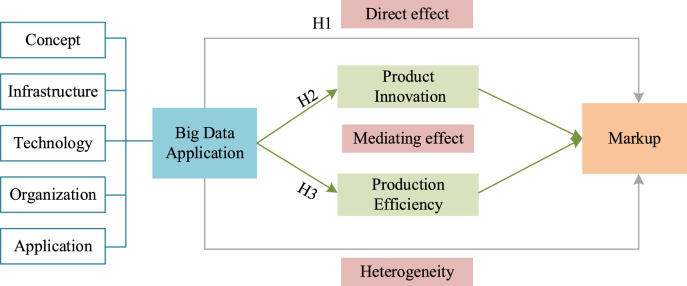
Theoretical analytical framework.

This study aims to elucidate the causal mechanism through which big data applications affect firms’ price markups. Accordingly, a theoretical analytical framework is constructed, as illustrated in Fig. 1.

### Theoretical model

This paper extends the theoretical model proposed by Antoniades^32^ and constructs a heterogeneous firm model with variable price markups to analyze the impact of enterprise big data applications on price markups and their underlying mechanisms.

#### Consumers

Consider a closed economy where consumer preferences are as follows:

1 $$U = q_{0}^{c} + \alpha \mathop \smallint \limits_{i \in \Omega } q_{i}^{c} di + \beta \mathop \smallint \limits_{i \in \Omega } z_{i} q_{i}^{c} di - \frac{1}{2}\gamma \mathop \smallint \limits_{i \in \Omega } \left( {q_{i}^{c} } \right)^{2} di - \frac{1}{2}\eta \left\{ {\mathop \smallint \limits_{i \in \Omega } q_{i}^{c} di} \right\}^{2}$$

Here, $q_{0}^{c}$ represents the consumer’s consumption of the numeraire good, and $q_{i}^{c}$ denotes the consumer’s consumption of the heterogeneous product $i$. The parameters $\alpha> 0$ and $\eta> 0$ together measure the substitutability between the numeraire good and heterogeneous products. $\beta> 0$ captures the consumer’s baseline preference for innovative products, and $\gamma> 0$ reflects the degree of preference for heterogeneous products. The set ${\Omega }$ represents all available heterogeneous products. Unlike Antoniades^32^, this paper introduces the variable $z_{i}$ to represent the innovativeness of a product, quantifying its level of innovation. Assume the price of the numeraire good is normalized to 1, and let $p_{i}$ be the price of product i. Solving the utility maximization problem yields the inverse demand function of the consumer:

2 $$p_{i} = \alpha + \beta z_{i} - \gamma q_{i}^{c} - \eta Q^{c}$$

where $Q^{c} = \mathop \smallint \limits_{{i \in {\Omega }}} q_{i}^{c} di$ represents the total consumption of heterogeneous products in the market. Considering the country size ${\mathrm{L}}$, the total demand function for product $i$ can be derived:

3 $$q_{i} = Lq_{i}^{c} = \frac{\alpha L}{{\eta N + \gamma }} - \frac{L}{\gamma }p_{i} + \frac{L\beta }{\gamma }z_{i} + \frac{\eta NL}{{\eta N + \gamma }}\overline{p} - \frac{\eta NL\beta }{{\eta N + \gamma }}\overline{z}$$

Here, ${\mathrm{N}} = { }\left| {{\Omega }^{*} } \right|$ denotes the number of product varieties, while $\overline{p} = \frac{1}{N}\mathop \smallint \limits_{{i \in {\Omega }^{*} }} p_{i} \,di$ and $\overline{z} = \frac{1}{N}\mathop \smallint \limits_{{i \in {\Omega }^{*} }} z_{i} di$ represent the average price and average innovation level of the products, respectively.

#### Firms

Assume labor is the only factor of production. Firms must pay a fixed cost $f_{E}$ to enter the market, and their marginal cost $c$ follows a distribution $G\left( c \right)$ with support $\left[ {0,c_{m} } \right]$. Only firms with a marginal cost below the threshold $c_{D}$ can survive and operate in the market. Different from the setting in Yu et al.^10^, this paper introduces a unit cost coefficient $\delta$ for product innovation into the model. The total cost function of a firm can be expressed as:

4 $$TC_{i} = q_{i} c_{i} + q_{i} \delta \frac{{z_{i} }}{\kappa } + \theta (z_{i} )^{2} + C\left( a \right)$$

Here,$q_{i} c_{i}$ represents the unit variable production cost, $q_{i} \delta \frac{{z_{i} }}{{\upkappa }}$ denotes the unit product innovation cost,$\delta$ is the cost coefficient for product innovation, and $\kappa$ represents the firm’s innovation efficiency. $(z_{i} )^{2}$ represents the fixed cost of product innovation for the firm, which is convex to reflect the increasing difficulty of product innovation.$\theta$ captures the heterogeneity in product innovation at the industry level. $a$ denotes big data application, and $C\left( a \right)$ represents the fixed cost paid by the firm for big data application, satisfying $C{ prime }\left( a \right)> 0$.${\upkappa } = {\mathrm{f(a)}}$ indicates the impact of big data application on innovation efficiency, satisfying $f^{\prime}\left( a \right)> 0$ and $f^{\prime\prime}\left( a \right) < 0$, reflecting diminishing marginal returns.

The basic principle underlying the assumption that $f\prime \left( a \right)> 0$ rests on several points. Big data is typically characterized by its “3V” features: Variety, Velocity, and Volume. The key to big data applications lies in effectively integrating and leveraging these characteristics, especially Variety and Velocity^33^. Specifically, by aggregating multi-source customer data, firms can gain in-depth insights into customer needs and preferences, thereby providing a basis for developing appropriate new product strategies.Simultaneously, by utilizing real-time data integration capabilities, firms can base their decisions on the most up-to-date evidence rather than historical trends, thus obtaining more timely and insightful information to formulate suitable new product strategies.

Additionally, by broadening the scope of a firm’s search for existing knowledge, big data applications facilitates the integration of cross-domain knowledge to form new technologies and provides critical support for incremental process improvements based on in-depth operational data^26^. Furthermore, data analysis techniques can effectively connect internally dispersed invention groups with existing technologies, promoting the integration of external knowledge and technology into internal innovation processes, thereby driving distributed innovation within firms^27^.

The rationale for proposing $f^{\prime\prime}\left( a \right) < 0$ is primarily based on the phenomenon of diminishing marginal returns in big data applications:① as innovation advances towards higher complexity and disruptiveness, technological difficulty and uncertainty increase exponentially, and the role of big data applications in improving innovation efficiency tends to diminish; ②as big data applications shift from early-stage extraction of key explicit value to later-stage refined and marginal data collection, the cost of information extraction rises significantly, while the additional insights obtainable become increasingly limited, collectively leading to a decline in marginal returns.

Following the approach of Melitz and Ottaviano^34^, at the threshold $c_{D}$ , ${\mathrm{q}}\left( {{\mathrm{c}}_{{\mathrm{D}}} {,0}} \right){ = 0}$ and ${\mathrm{p}}\left( {{\mathrm{c}}_{{\mathrm{D}}} {,0}} \right){\text{ = c}}_{{\mathrm{D}}}$, leading to the derivation: $c_{D} = \frac{{\alpha \gamma + \eta N\overline{p} - \eta N\beta \overline{z} }}{\eta N + \gamma }$,Price and demand can be expressed as:

5 $$p\left( {c,z} \right) = \frac{1}{2}\left[ {c_{D} + c} \right] + \frac{1}{2}\left( {\beta + \frac{\delta }{f\left( a \right)}} \right)z$$

6 $$q\left( {c,z} \right) = \frac{L}{2\gamma }\left[ {c_{D} - c} \right] + \frac{L}{2\gamma }\left( {\beta - \frac{\delta }{f\left( a \right)}} \right)z$$

Therefore, the profit function is:

7 $$\pi \left( {c,z} \right) = \frac{L}{4\gamma }\left[ {\left( {c_{D} - c} \right) + \left( {\beta - \frac{\delta }{f\left( a \right)}} \right)z} \right]^{2} - \theta z^{2} - C\left( a \right)$$

The firm’s optimal product innovation level is:

8 $$z^{*} = \lambda \left( {c_{D} - c} \right),\lambda = \frac{{L\left( {\beta - \frac{\delta }{f\left( a \right)}} \right)}}{{4\theta \gamma - L(\beta - \frac{\delta }{f\left( a \right)})^{2} }}$$

To ensure ${\mathrm{z}}^{*}> 0$, the conditions $4\theta \gamma> L(\beta - \frac{\delta }{f\left( a \right)})^{2}$ and $\beta> \frac{\delta }{f\left( a \right)}$ must be satisfied. This is because consumers’ willingness to pay for innovative products must be higher than the marginal cost of the firm’s product innovation. Substituting ${\mathrm{z}}^{*}$ into the original equation yields the simplified expressions for price, demand, and profit:

9 $$p\left( c \right) = \frac{1}{2}\left( {c_{D} + c} \right) + \frac{1}{2}\left( {\beta + \frac{\delta }{f\left( a \right)}} \right)\lambda \left( {c_{D} - c} \right)$$

10 $$q\left( c \right) = \frac{L}{2\gamma }\left[ {1 + \left( {\beta - \frac{\delta }{f\left( a \right)}} \right)\lambda } \right]\left( {c_{D} - c} \right)$$

11 $$\pi \left( c \right) = \frac{L}{4\gamma }\left[ {1 + \left( {\beta - \frac{\delta }{f\left( a \right)}} \right)\lambda } \right](c_{D} - c)^{2} - C\left( a \right)$$

#### Big data applications and price markups

(1) Big data applications and firm price markups.

With reference to the research by Antoniades^32^, the marginal cost of an enterprise is defined as $MC = c + \frac{\delta }{f\left( a \right)}z$. The price markup of the enterprise is then defined as:

12 $$\mu \left( c \right) = p\left( c \right) - MC = \frac{1}{2}\left[ {1 + \left( {\beta - \frac{\delta }{f\left( a \right)}} \right)\lambda } \right]\left( {c_{D} - c} \right)$$

It is evident that $\frac{{\partial {\upmu }\left( {\mathrm{c}} \right)}}{{\partial {\mathrm{a}}}}> 0$ (see Appendix 1 for detailed derivation). Steinberg^35^ pointed out that enterprises can accurately gauge consumers’ willingness to pay and preferences through big data analytics, thereby implementing personalized dynamic pricing strategies to more effectively capture consumer surplus. Eeckhout and Veldkamp^36^ further suggested that data analysis enhances price markup capabilities by reducing risks faced by enterprises, promoting business scale expansion, and optimizing product structures. Thus, the following hypothesis is proposed:

H1: Big data applications contribute to enhancing enterprise price markups.

(2) Big data application, product innovation, and firm price markup.

By taking the derivative of product innovation with respect to big data application, we obtain:

13 $$\frac{\partial z * }{{\partial a}} = \frac{{L\delta \left( {c_{D} - c} \right)\left( {4\theta \gamma + Lx^{2} } \right)f^{\prime}\left( a \right)}}{{(4\theta \gamma - Lx^{2} )^{2} f(a)^{2} }}$$

It can be deduced that $\frac{{\partial z_{i}^{*} }}{\partial a}> 0$ (see Appendix 1 for detailed derivation). The result indicates that firms’ application of big data can effectively promote product innovation.

Firstly, knowledge is cumulative and serves as a key driver of technological progress. Data, as a byproduct of production activities, can become an important source of knowledge accumulation in the “learning-by-doing” process^37^. Secondly, the scale and diversity of big data expand the boundaries of firms’ information search, while big data platforms significantly enhance information processing efficiency. The integration and fusion of multi-source heterogeneous data can substantially accelerate the process of product innovation. Furthermore, the ability to accumulate and analyze large-scale, diverse, and high-velocity data enhances firms’ predictive capabilities^26^, thereby reducing the uncertainty and R&D costs associated with product innovation. Some empirical studies also support these conclusions (e.g^38^).

Further, from $\frac{d\mu }{{dz_{i}^{*} }} = \frac{1}{2}\left( {\beta - \frac{\delta }{f\left( a \right)}} \right)$ , and based on the earlier assumption that $\beta> \frac{\delta }{f\left( a \right)}$, it follows that $\frac{d\mu }{{dz_{i}^{*} }}> 0$.On one hand, product innovation enhances product uniqueness and perceived value, making consumers willing to pay a higher price. On the other hand, the differentiation advantage resulting from product innovation reduces direct competition and grants firms stronger pricing power^39^. Therefore, it can be inferred that $\frac{\partial \mu }{{\partial z_{i}^{*} }} \times \frac{{\partial z_{i}^{*} }}{\partial a}> 0$. Based on the above analysis, this study proposes research hypothesis H2:

Hypothesis H2: Big data application enhances firms’ price markup by enabling product innovation.

(3) Big data application, production efficiency, and firm price markup.

The firm’s price markup is expressed as: $\mu = \frac{1}{2}c_{D} + \frac{1}{2}z\left[ {\beta - \left( {\frac{c}{z} + \frac{\delta }{f\left( a \right)}} \right)} \right]$. Here, $\frac{c}{z} + \frac{\delta }{f\left( a \right)}$ represents the sum of variable costs and innovation costs per unit product, which is used to calculate the total marginal cost of production and product innovation. The impact of big data application on production efficiency is given by:

14 $$\frac{{d\left( {\frac{c}{z} + \frac{\delta }{f\left( a \right)}} \right)}}{da} = - \frac{\delta f\prime \left( a \right)}{{[f\left( a \right)]^{2} }} < 0$$

The derivation above indicates that the application of big data can effectively reduce the unit cost of products, thereby enhancing production efficiency. By integrating real-time data from production, inventory, and finance, big data applications breaks down information barriers between departments, creating a unified and transparent data view. This helps reduce trust issues arising from information asymmetry and optimizes internal communication and collaboration^6^. Additionally, enterprises can leverage big data to efficiently analyze vast amounts of market information, accurately identify changes in demand, and provide timely and accurate support for R&D and strategic decision-making^25^. As a general-purpose technology, big data applications can automate low-skill tasks and assist in high-skill decision-making, promoting a shift in the workforce structure toward higher skills and comprehensively improving productivity^40^. Brynjolfsson et al.^41^ found that large U.S. firms adopting data-driven decision-making exhibited 5%–6% higher productivity. Tambe^6^ also noted that publicly traded companies investing in big data technologies experienced approximately 3% productivity gains.Furthermore, from $\frac{d\left( \mu \right)}{{d\left( {\frac{c}{z} + \frac{\delta }{f\left( a \right)}} \right)}} = - \frac{1}{2}z> 0$, it follows that: $\frac{d\left( \mu \right)}{{d\left( {\frac{c}{z} + \frac{\delta }{f\left( a \right)}} \right)}} \times \frac{{d\left( {\frac{c}{z} + \frac{\delta }{f\left( a \right)}} \right)}}{da}> 0$.Due to the reduction in unit costs resulting from improved production efficiency, firms’ profit margins expand, thereby increasing their price markup. Thus, research hypothesis H3 is proposed:

H3: Big data application drives a significant improvement in firms’ production efficiency, which in turn enhances their price markup.

## Research design

### Model specification

The two-way fixed effects model can simultaneously control for unobservable factors at both the firm and time levels that do not change over time, thereby more effectively mitigating omitted variable bias. To examine the relationship between big data application and firm price markup, this paper constructs a two-way fixed effects model:

15 $$Markup_{it} = \alpha + \beta LnBigData_{it} + \gamma X_{it} + \mu_{i} + \lambda_{t} + \in_{it}$$

Here,${\mathrm{Markup}}_{{{\mathrm{it}}}}$ represents the price markup of firm ${\mathrm{i}}$ in year $t$. ${\mathrm{LnBigData}}_{{{\mathrm{it}}}}$ denotes the level of big data application of firm ${\mathrm{i}}$ in year $t$. ${\mathrm{X}}_{{{\mathrm{it}}}}$ is a set of time-varying control variables used to alleviate omitted variable bias.${\mu }_{i}$ represents firm fixed effects, controlling for all time-invariant firm characteristics.${\lambda }_{t}$ denotes time fixed effects, controlling for macroeconomic shocks common to all firms. ${\epsilon }_{it}$ is the random error term. $\beta$ indicates the impact of an increase in a firm’s big data application level on its price markup, after controlling for other variables, firm individual effects, and time effects.

This study faces endogeneity issues arising from reverse causality and omitted variables: on the one hand, an increase in a firm’s price markup may conversely incentivize it to expand the scale of big data technology adoption; on the other hand, unobservable factors (such as managerial capabilities or industry-specific technological shocks) may simultaneously influence both a firm’s level of big data adoption and its markup decisions. To effectively mitigate these endogeneity biases, this paper selects the number of post offices per million people in each city in 1984 as an instrumental variable. This variable satisfies both relevance and exogeneity conditions: as a core component of traditional information infrastructure, the density of post offices significantly shaped the early coverage levels of landline telephones and the internet in various regions, potentially influencing current levels of big data technology adoption among firms through historical path dependency. At the same time, since its functions have been largely replaced by modern communication technologies, this variable has no direct transmission channel to affect contemporary firms’ price markups, and it is unlikely to influence current pricing behavior through other unobservable factors, thereby satisfying the exclusion restriction.Given that the sample consists of a balanced panel dataset, directly using this variable in a fixed-effects model may yield insufficient variation. Therefore, this paper constructs an interaction term between the 1984 city-level number of post offices per million people and the previous year’s national internet user count as the instrumental variable for big data application.

### Indicator description

#### Explained *v*ariable

This paper adopts the method proposed by Loecker & Warzynski^42^ to estimate firm-level price markups based on a translog production function. This approach offers the following advantages: First, it directly estimates the deviation between price and marginal cost from the cost side, aligning closely with the theoretical definition. Second, it relies solely on financial statement data, making it highly operable. Third, the method has gained widespread recognition in academia, ensuring the comparability and reliability of the research results. The core idea involves first estimating the output elasticity of variable factors through the production function, then combining this with the actual expenditure share of the factor to calculate the markup ratio as the ratio of the two. This paper employs the two-stage estimation method proposed by Ackerberg et al.^43^ to obtain the output elasticity of intermediate inputs under the translog form. The detailed derivation process and data used are provided in Appendix 2.

#### Core explanatory variable

The core explanatory variable in this paper is the level of big data application by firms, which refers to the process of using technologies such as data collection, storage, cleaning, analysis, and mining to process massive, multi-source, and rapidly growing data, extract valuable information, and apply it to practical business operations to optimize decision-making and create value^21^. Existing studies often use the core variable method or questionnaire surveys for measurement. However, the former struggles to comprehensively cover the big data framework, while the latter is susceptible to subjective biases. Corporate annual reports, which cover all listed companies, serve as an ideal data source. Zhang et al.^29^ measured the level of big data application by counting the frequency of big data-related keywords in annual reports, but this method is prone to text noise interference. In recent years, large language models (LLMs) have been widely used as efficient text analysis tools, as seen in studies by Li et al.^30^ and Fang et al.^31^, providing new technical pathways and research possibilities for measuring big data application levels from corporate annual reports.

To more systematically capture the overall framework of corporate big data application and cover as many Chinese listed companies as possible, this paper draws on the research of Fang et al.^31^ by constructing a lexicon of big data application terms and leveraging large language models to mine text from listed companies’ annual reports, thereby constructing a measure of corporate big data application. First, based on the principles of scientific rigor, systematicity, and operability, this study constructs a lexicon of 30 keywords across five dimensions—conceptual, foundational, technological, organizational, and application layers—using text data from listed companies’ annual reports, as shown in Table 1.

**Table 1:** Big data application lexicon.

Dimension	Keywords	Partial source indicators
Conceptual Layer	Big data, massive data, data-driven, data asset, data element, data analysis	^29,44^
Foundational layer	Data center, data warehouse, data lake, data middle platform, cloud platform, computing center	^44,45^
Technical layer	Data mining, data visualization, hadoop, spark, machine learning, deep learning	^6,44^
Organizational layer	Data scientist, data analyst, data engineer, data architect, chief information officer, chief digital officer	^3,27^
Application layer	User profiling, intelligent recommendation, business intelligence, precision marketing, real-time personalization, intelligent customer service	^38,44^

Hadoop and Spark are widely used distributed computing frameworks in the field of big data, designed for processing massive datasets.

Second, a Python program is used to batch extract sentences related to the above lexicon from the annual reports. Subsequently, by calling the Alibaba Cloud API interface via Python, the Qwen-Turbo model is employed to intelligently analyze the extracted sentences, completing the following two judgments sequentially: (i) identifying whether the sentences contain keywords from the big data application lexicon and determining their respective dimensions; and (ii) judging whether the sentences genuinely reflect the specific circumstances of the firm’s implementation of big data applications. The judgment results are output as “yes” or “no.” The Qwen-Turbo model is selected primarily for two advantages: first, it excels in Chinese language understanding and generation, making it more suitable for processing Chinese text compared to large models like GPT and Claude; second, it supports an ultra-long context window, enabling effective handling of complex semantic judgment tasks that rely on extensive contextual information.

Finally, the corporate big data application index (LnBigData) is constructed by taking the natural logarithm of the total frequency of “yes” judgments across all dimensions for each firm, plus one. The keywords involved in this study and their specific definitions are provided in Table 1. Additionally, the validity of the corporate big data application indicator is comprehensively tested from multiple perspectives, including model performance comparison, comparison of corporate big data-related indicators, and verification against real-world scenarios. Detailed content is provided in Appendix 3.

#### Control variables

Based on existing literature^46,47^, this study selects the following variables as controls: firm size (Size, measured by the number of employees, following the approach of Bloom & Van Reenen^48^ and Ghasemaghaei & Calic^33^),growth (Growth, measured as the growth rate of operating revenue, i.e., (current period operating revenue—previous period operating revenue) / previous period operating revenue); leverage ratio (Lev, measured as the ratio of total liabilities to total assets); cash flow (Cashflow, measured as the ratio of net cash flow from operating activities to total assets); board size (Board, measured as the natural logarithm of the number of board members); CEO duality (Dual, a dummy variable that equals 1 if the roles of chairman and general manager are held by the same person, and 0 otherwise); and firm age (FirmAge, measured as the natural logarithm of the number of years since establishment). Descriptive statistics for the main variables are presented in Table 2. We performed a 1% winsorization on the data to reduce the potential impact of outliers on the analytical results, ultimately obtaining 19,555 valid enterprise observations.

**Table 2:** Descriptive statistics.

Variable	Obs	Mean	Std. dev	Min	Max
Markupm	19,555	1.2287	0.1990	0.5683	1.9529
LnBigdata	19,555	1.3856	1.0169	0.0000	5.9661
Size	19,555	0.5640	1.0406	0.0048	7.1736
Growth	19,555	0.1422	0.3537	− 0.6712	3.8082
Lev	19,555	0.4216	0.1995	0.0274	0.9901
Cashflow	19,555	0.0489	0.0669	− 0.2325	0.2788
Board	19,555	2.1067	0.2017	1.0986	2.8332
Dual	19,555	0.3138	0.4641	0.0000	1.0000
FirmAge	19,555	2.9729	0.3310	1.0986	3.6376

### Data sources

This study uses Chinese listed companies as the sample, with a research period spanning from 2002 to 2023. The explained variable is the firm’s price markup, and the data are sourced from the CSMAR database. The core explanatory variable is corporate annual report information, obtained from the official website of Sina Finance. Control variables include corporate financial and governance data, all of which are sourced from the CSMAR database. Additionally, the patent data used in this study are obtained from the IncoPat patent database, and the data for the instrumental variables are sourced from the National Bureau of Statistics database. To ensure the rigor and reliability of the empirical research, samples that were subject to Special Treatment , had severe data deficiencies, or exhibited data quality issues were excluded.

## Empirical results and analysis

### Baseline regression

Table 3 reports the baseline regression results on the impact of big data application on corporate price markups. Column (1) only controls for firm and year fixed effects without including other control variables. The results show that the estimated coefficient of LnBigdata is significantly positive at the 1% level, indicating that big data application helps enhance corporate price markups. Column (2) further incorporates other control variables based on Column (1), and it is found that big data application still has a significant positive impact on corporate price markups, remaining significant at a high level of significance. By comparing the results of Column (2) with Column (1), it can be observed that after controlling for other variables, the regression coefficient of big data application changes slightly, but its significance and positive effect remain consistent, further indicating that big data application has a robust promoting effect on corporate price markups. Unlike Eeckhout and Veldkamp^7^, who confirmed data-driven price markups at the macro level, this study examines the impact of big data on corporate price markups at the micro level, representing a further extension of the existing literature.

**Table 3:** Baseline regression.

	OLS		2SLS	
	(1)	(2)	(3)	(4)
	Markup	Markup	LnBigdata	Markup
LnBigdata	0.0132***	0.0125***		0.1750***
	(0.0024)	(0.0024)		(0.0368)
Ln(Post × Internet_t-1_)			0.1282***	
			(0.0185)	
Controls	NO	YES	YES	YES
Firm FE	YES	YES	YES	YES
Year FE	YES	YES	YES	YES
Observations	19,555	19,555	8590	8590
R-squared	0.7947	0.7975		
Kleibergen-Paap rk LM statistic			47.16 [0.0000]	
Kleibergen-Paap rk Wald F statistic			47.99 {16.38}	

Robust standard errors clustered at the firm level are reported in parentheses;***, **, and * denote statistical significance at the 1%, 5%, and 10% levels, respectively. The same applies hereafter.

The two-stage least squares (2SLS) estimation results show that the Kleibergen-Paap rk LM statistic is 47.16 significant at the 1% level, rejecting the null hypothesis of “underidentification of instrumental variables.” The Kleibergen-Paap rk Wald F statistic is 47.99, far exceeding the Stock-Yogo critical value (16.38) at the 10% level, indicating no weak instrument problem. These results confirm the validity of the instrumental variables, and the 2SLS estimation effectively mitigates endogeneity issues. Meanwhile, the coefficient of big data application (LnBigdata) is significantly positive at the 1% level, consistent with the baseline regression results, supporting the conclusion that big data application promotes corporate price markups. Therefore, research hypothesis H1 is validated.

### Robustness tests

#### Changing the instrumental variable

To further enhance the robustness of the conclusions, this study selects the “Broadband China” pilot policy as an alternative instrumental variable for testing. This policy, implemented by the Ministry of Industry and Information Technology and the National Development and Reform Commission in three batches across 120 cities from 2014 to 2016, aimed to improve broadband coverage, internet speed, and user scale to promote economic and social development. From the perspective of instrumental variable validity, better information infrastructure helps reduce the costs for enterprises to deploy and operate big data applications, thereby encouraging their adoption. Moreover, the level of urban communication infrastructure is largely determined by macro-level policies, making it exogenous to individual firms. The results in Table 4 show that, after changing the instrumental variable, the estimated coefficients of the core variables remain statistically significant and consistent in sign with the baseline regression. This further confirms the positive effect of big data applications on firms’ price markups, indicating the reliability of the baseline conclusions.

**Table 4:** Estimation results with alternative instrumental variable.

	(1)	(2)
	LnBigdata	Markup
Broadband China	0.3719***	
	(0.0157)	
LnBigdata		0.0379***
		(0.0092)
Controls	YES	YES
Firm FE	YES	YES
Year FE	YES	YES
Observations	17,559	17,559
Kleibergen-Paap rk LM statistic	529.36 [0.0000]	
Kleibergen-Paap rk Wald F statistic	560.28 {16.38}	

#### Replacing the explained variable

To enhance the robustness of the estimation results, this study employs both the Levinsohn-Petrin method and a two-way fixed effects model to estimate the production function, thereby obtaining the corresponding labor elasticity coefficients. Based on this, the price markup indicators (MarkupLP and MarkupFE) for firms are constructed by dividing the labor elasticity coefficients by the share of labor costs in total revenue. Table 5 reports the estimation results after replacing the explained variable, where the coefficient of LnBigdata is significantly positive at the 1% level, indicating that the baseline estimation results are highly robust.

**Table 5:** Estimation results after replacing the explained variable.

	(1)	(2)
	MarkupLP	MarkupFE
LnBigdata	0.0084**	0.0092**
	(0.0034)	(0.0037)
Controls	YES	YES
Firm FE	YES	YES
Year FE	YES	YES
Observations	16,552	16,552
R-squared	0.8748	0.8748

#### Replacement of the core explanatory variable

Since the construction of the core explanatory variable, big data application (LnBigData), is based on textual data from listed companies’ annual reports, it may be influenced by variations in the quality and content of textual disclosures. To verify the robustness of the baseline regression results, this study adopts the following three methods to replace the core explanatory variable for re-examination: ① Drawing on the approaches of Tambe^6^ and Wu et al.^27^, this study crawls recruitment information published by listed companies on human resource platforms such as “51job,” “BOSS Zhipin,” and “Zhaopin.” Positions with job titles or descriptions containing keywords such as “big data,” “data analysis,” and “data mining” are screened, and the natural logarithm of their quantity (LnBDJob) is used as a measurement indicator. ② Following Ridder^17^, this study screens patents and technologies containing keywords such as “data management system” and “software” based on the detailed items of intangible assets in the notes to financial statements. The ratio of the total amount of big data-related intangible assets to the total intangible assets (IntangibleAssetsBD) is used as a proxy variable. ③ Based on the digital economy patent database constructed from the IncoPat patent database, this study extracts digital patents related to technologies such as big data for each enterprise annually. The total number of patents is counted, and its natural logarithm (LnBDPatent) is used as a proxy variable for the level of big data application by enterprises.

Table 6 presents the estimation results using the three alternative variables mentioned above. The estimated coefficients of the core explanatory variables remain consistently positive and highly significant at the 1% statistical level, which fully confirms the robustness of the baseline regression conclusions.

**Table 6:** Estimation results with replacement of core explanatory variables.

	(1)	(2)	(3)
	Markup	Markup	Markup
LnBDJob	0.0022*		
	(0.0012)		
IntangibleAssetsBD		0.0648**	
		(0.0269)	
LnBDPatent			0.4551*
			(0.2487)
Controls	YES	YES	YES
Firm FE	YES	YES	YES
Year FE	YES	YES	YES
Observations	16,960	33,296	42,751
R-squared	0.8259	0.7450	0.6906

#### Other robustness tests

This paper also conducted the following robustness tests: ① Using the five sub-dimensions of enterprise big data application as the core explanatory variables; ② Adopting measurement methods from classical literature; ③ Incorporating control variables at the city and industry levels on the basis of the baseline model, including industry concentration (measured by the HHI index), regional economic development level, and government technology expenditure; ④ Adjusting clustered standard errors to both industry and city levels to enhance the reliability of statistical inference; ⑤ Applying winsorization at the top and bottom 1% to all continuous variables in the model to mitigate the potential impact of outliers on estimation results; ⑥ Adding an IT industry dummy variable to control for industry heterogeneity; ⑦ Introducing a pandemic time dummy variable to control for the impact of the pandemic shock on the results; ⑧ Simultaneously controlling for city and industry fixed effects in the model to capture time-invariant regional and industry characteristics. Tables A3 to A5 in Appendix 4 report the estimation results after replacing the core explanatory variables. The estimated coefficients for big data application remain significantly positive at the 1% level, consistent with the baseline conclusion, indicating strong robustness of the original estimation results. This further confirms that big data has a significant positive effect on enterprise price markup.

## Mechanism analysis

In the mechanism testing section, this paper focuses on examining the impact of enterprise big data application on product innovation and production efficiency. This choice is primarily based on two considerations: on one hand, traditional three-step mediation effect methods suffer from endogeneity issues, which may affect the reliability of estimation results; on the other hand, existing research has sufficiently demonstrated the significant influence of product innovation and productivity on firms’ price markup (Helpman & Niswonger, 2022^39^;). The following model is constructed:

16 $$mechanism_{it} = \alpha + \beta LnBigData_{it} + \gamma X_{it} + \mu_{i} + \lambda_{t} + \in_{it}$$

Here, $mechanism_{it}$ refers to the mechanism variables tested in this study, while other control variables remain consistent with the baseline regression. Firm-level clustered robust standard errors are employed in the mechanism testing process to control for heteroscedasticity and serial correlation at the individual level.

### Product innovation

By leveraging big data applications, enterprises can gain more precise insights into consumer needs, effectively drive product innovation, and achieve rapid iteration and experience optimization. By identifying consumer preferences, companies can strategically add features that users are willing to pay a premium for, while reducing elements that are not recognized by the market or have low demand^35^, thereby enhancing the product’s premium pricing capability. Furthermore, big data applications not only optimizes the innovation process through high-quality information but also serves as a core driver of innovation, fostering the development of new digital products and services (Niebel et al., 2018).

With reference to the study by Niebel et al. (2018), this paper measures firms’ product innovation capability from three dimensions: the degree of product innovation, R&D expenditure intensity, and R&D efficiency. Specifically, the level of product innovation (LnProductInno) is measured by counting the number of patent applications containing keywords such as “equipment,” “device,” “machine,” and “instrument” in the firm’s name, while excluding keywords such as “method,” “process,” “software,” “program,” and “algorithm,” and then taking the natural logarithm. This directly reflects the firm’s ability to introduce or improve products. Furthermore, since invention patents cover both product and process innovations, this paper additionally uses the number of utility model patent and design patent applications as a supplementary indicator to measure firms’ product innovation performance. This indicator is specifically expressed as the natural logarithm of the total number of these two types of patent applications plus one (LnProductInnoUMP&DP). R&D expenditure intensity (R&D Intensity) is measured as the ratio of R&D expenditure to total assets, reflecting the level of resources a firm invests in innovation activities. R&D efficiency (R&D Efficiency) is calculated as ln(1 + number of patent applications)/ln(1 + R&D expenditure). This indicator considers both innovation input and output, measuring the efficiency of a firm’s utilization of innovation resources. Panel A of Table 7 reports the test results for the product innovation mechanism. The results show that the coefficient of big data application (LnBigdata) is significantly positive at the 1% level, indicating that big data application significantly enhances firms’ product innovation. Therefore, research hypothesis H2 is supported.

**Table 7:** Mechanism tests: product innovation and production efficiency.

	(1)	(2)	(3)	(4)
Panel A:product innovation	LnProductInno	LnProductInno UMP&DP	R&D Intensity	R&D Efficiency
LnBigdata	0.0360**	0.0290*	0.0010***	0.0034***
	(0.0181)	(0.0165)	(0.0002)	(0.0010)
Observations	14,619	18,427	21,748	19,965
R-squared	0.8235	0.8203	0.8900	0.7324
Panel B:production efficiency	TFP_LP	TFP_OP	LaborProductivityⅠ	LaborProductivityⅡ
LnBigdata	0.0353***	0.0538***	0.0172**	0.0206**
	(0.0087)	(0.0093)	(0.0085)	(0.0091)
Observations	19,864	19,864	21,153	19,479
R-squared	0.8993	0.9189	0.8932	0.8822
Controls	YES	YES	YES	YES
Firm FE	YES	YES	YES	YES
Year FE	YES	YES	YES	YES

### Production efficiency

Data-driven decision-making can significantly enhance corporate productivity by reducing information uncertainty and asymmetry^27^. The improvement in productivity further lowers the marginal cost per unit of product, enabling firms to respond more flexibly to changes in market demand, thereby effectively strengthening their price markup capability^17^.

In measuring corporate production efficiency, this study adopts total factor productivity (TFP) as the core evaluation metric, as it comprehensively reflects the contributions of various production factors and captures the combined effects of both “technological progress” and “efficiency improvement.” When estimating corporate TFP, this study draws on the approach of Levinsohn and Petrin^49^, employing the classic micro-level LP method (TFP_LP), supplemented by the OP method (TFP_OP)^50^ to enhance the robustness of the results. Additionally, labor productivity is used as a proxy for production efficiency, drawing on the measurement methods of Babina et al.^51^. Specifically, two approaches are adopted: “the ratio of operating revenue to total number of employees (LaborProductivityⅠ)” and “the logarithm of (sales revenue + change in inventory) divided by the number of employees (LaborProductivityⅡ).” Panel B of Table 7 reports the estimation results for the production efficiency mechanism. The results show that the coefficient of LnBigdata is significantly positive at the 1% level, indicating that big data application significantly enhances corporate production efficiency. Therefore, research hypothesis H2 is validated.

Furthermore, this study employs a three-stage regression model to examine the mediating pathways, and the related results provide additional support for the robustness of the main analysis. To more directly verify the significance of the mediating effects and quantify their magnitude, we systematically report the results of the Sobel test and the Bootstrap method in Appendix 5. The findings indicate that the application of big data can significantly enhance firms’ markup rates through two channels: product innovation and production efficiency.

## Heterogeneity analysis

Begenau et al.^5^ point out that digital technology development tends to disproportionately benefit a small number of firms, leading to increasing heterogeneity among enterprises. The underlying reason is that, in most cases, for firms to benefit from technological innovation, they must effectively integrate new technologies with their existing capabilities or assets. Additionally, the external institutional environment is also a crucial factor influencing corporate behavior and performance^46^. Therefore, in the process of big data applications influencing firms’ price markups, does a similar heterogeneity phenomenon exist? Based on the TOE framework , the adoption and application of new technologies by firms are influenced by a combination of technological, organizational, and environmental factors. Following this framework, this study systematically examines the heterogeneous factors affecting the relationship between big data applications and price markups from three dimensions: technology, organization, and environment.

### Organizational level

At the organizational level, this study focuses on firm size and workforce skill level. Literature suggests that IT-specific investments exhibit “scale bias,” meaning that intangible assets such as IT equipment and software can enhance firm productivity and market share, with this effect being more pronounced in larger firms^28^. Larger firms typically possess more advanced information infrastructure, more specialized technical talent, and more mature marketing networks. These complementary resources help improve the efficiency of big data technology applications^5^. Meanwhile, a high-skilled workforce generally possesses broader cross-disciplinary knowledge, stronger data modeling and analytical capabilities, and richer problem-solving experience. Such specialized resources significantly enhance the development and application of big data technologies. In this study, firms with total assets above the annual industry median are defined as large-scale firms (assigned a value of 1, otherwise 0), and the ratio of technical personnel to total employees is included as an interaction term in the analysis. ( Our theoretical basis for categorizing the ‘proportion of technical personnel’ under the organizational dimension, rather than the technological dimension, is as follows: In the TOE framework, the technology dimension typically refers to the firm’s existing technology stock and the characteristics of the technology itself, which are relatively objective environmental and tool sets. The organizational dimension focuses on the internal resource allocation, structural arrangements, and capability building that a firm undertakes to adopt and apply technology, reflecting proactive agency. The ‘proportion of technical personnel’ directly reflects the strategic allocation and long-term commitment to technological activities in the firm’s human resource structure. It belongs to the internal capability building that the organization undertakes to adapt to technology and is a key manifestation of ‘human capital’ within the organizational dimension. This differs from indicators in the technology dimension that focus more on describing objective conditions such as the maturity of technological infrastructure or technological compatibility.) Table 8 Panel A reports the estimation results. The coefficients of the interaction terms LnBigdata × Size and LnBigdata × WorkforceSkills are both significantly positive at the 1% level, indicating that compared to smaller firms or those with a lower proportion of technical personnel, larger firms and those with a higher proportion of technical personnel achieve more significant price markup improvements through big data applications.

**Table 8:** Heterogeneity analysis.

	(1)	(2)
	Markup	Markup
Panel A:organization		
LnBigdata × FirmSize	0.0103***	
	(0.0036)	
LnBigdata × WorkforceSkills		0.0020***
		(0.0003)
Observations	19,288	12,518
R-squared	0.8011	0.8081
Panel B:technology		
LnBigdata × LnDigitalTechnology	0.4257***	
	(0.1316)	
LnBigdata × Technology-Intensive		0.0381***
		(0.0058)
Observations	16,311	19,555
R-squared	0.7975	0.8006
Panel C:environment		
LnBigdata × DigitalBusinessEnv​	0.0073***	
	(0.0015)	
LnBigdata × Marketization		0.0081***
		(0.0012)
Observations	18,089	19,544
R-squared	0.7989	0.7988
Controls	YES	YES
Firm FE	YES	YES
Year FE	YES	YES

### Technological level

At the technological level, this study focuses on firms’ technological reserves and industry-level technology application. Strong technological reserves generally facilitate the penetration of big data technologies into higher-value application scenarios. Meanwhile, technology-intensive industries typically exhibit higher R&D investment, stronger innovation capabilities, and richer knowledge accumulation, giving them advantages in acquiring, integrating, and analyzing data resources. Based on the digital economy-related patent sub-database in the incoPat patent database, this study compiles and logarithmically transforms relevant patent data of Chinese listed firms to measure their technological reserves. Additionally, following the “CSRC 2012 Industry Classification Standard,” 12 industries, including information transmission, software, and information technology services, are defined as technology-intensive industries (assigned a value of 1, otherwise 0). The estimation results in Table 8 Panel B show that the coefficients of the interaction terms LnBigdata × LnDigitalTechnology and LnBigdata × Technology-Intensive are both significantly positive at the 1% level, indicating that compared to firms with weaker technological reserves or lower industry-level technology application, firms with stronger technological reserves or those in technology-intensive industries achieve more significant price markup improvements through big data applications.

### Environmental level

At the environmental level, this study focuses on the digital economy business environment and regional marketization level. The digital economy business environment refers to the external conditions on which digital firms rely for survival and development, encompassing policy support, legal norms, market mechanisms, and infrastructure. It not only provides institutional guarantees and data circulation rules for big data development but also determines its speed, quality, and security. The regional marketization level reflects the extent to which the market dominates resource allocation. Its improvement helps reduce institutional transaction costs, incentivizes firms to pursue product differentiation, and enhances market power, thereby improving firms’ price markup capabilities. In terms of variable measurement, a favorable business environment can effectively attract investment and entrepreneurial activities. Accordingly, this study uses the logarithm of the number of newly registered firms in the digital economy industry at the city level as a measure of the digital economy business environment, with data sourced from the Chinese Business Registration Database. Simultaneously, drawing on the research of Wang et al.^52^, a provincial-level comprehensive marketization index is constructed from multiple dimensions, including the relationship between the government and the market, the development of the non-state economy, the development of product and factor markets, the development of intermediary organizations, and the legal environment. Data for this index are obtained from the official database of the National Bureau of Statistics of China. The estimation results in Table 8 Panel C show that the interaction terms LnBigdata × DigitalBusinessEnv and LnBigdata × Marketization are both significantly positive at the 1% level, indicating that a favorable digital economy business environment and a higher regional marketization level can enhance the positive effect of big data applications on firms’ price markups.

## Research conclusions and policy implications

### Research conclusions

Deepening the application of big data in enterprises is a critical pathway to fully unleash the potential of data elements and empower businesses to enhance quality and efficiency. This study aims to explore the impact and mechanisms through which enterprise big data application influences price markups. First, building on the theoretical framework of Antoniades^32^, we develop a model of variable price markups for heterogeneous firms to theoretically analyze how big data application affects corporate cost markups. Second, we propose a text mining method based on large language models to analyze corporate annual reports, constructing a big data application indicator for enterprises. Using this indicator, we empirically examine the relationship between enterprise big data application and price markups through a two-way fixed effects model.

The findings indicate that the application of big data significantly enhances enterprises’ price markup capabilities. Mechanistically, big data application primarily boosts price markups by improving production efficiency and fostering innovation. Heterogeneity analysis further reveals that the impact of big data application on price markups varies significantly across organizational, technological, and environmental dimensions. Specifically, at the organizational level, the promotional effect of big data application on price markups is more pronounced in larger firms and those with a higher proportion of technical personnel. At the technological level, this effect is more substantial in enterprises with richer technological reserves and higher technological intensity. At the environmental level, enterprises located in regions with a more advanced digital economy, a better business environment, and a higher degree of marketization exhibit a more significant enhancement in price markups due to big data application. This study provides empirical evidence for understanding how big data shapes corporate competitiveness and market power, offering academic support and decision-making references for the formulation and optimization of big data-related policies.

### Policy implications

The findings of this study carry important policy implications. For local governments, since big data application significantly enhances corporate price markups, it is essential to actively promote the high-quality development of the big data industry. Specifically, efforts should first focus on strengthening top-level design and regional planning, establishing efficient and secure data circulation and transaction mechanisms, and continuously improving the digital infrastructure support system. Simultaneously, it is necessary to implement differentiated support policies, with particular attention to small and medium-sized enterprises, firms with a high proportion of low-skilled labor, and traditional industries. By providing digital training, technical support, and financial subsidies, the “digital divide” can be effectively bridged.

For enterprises, it is crucial to proactively integrate big data technology into production, operation, and innovation processes to fully leverage its core value in enhancing production efficiency and innovation capability. Moreover, enterprises of different sizes and types should develop differentiated and phased strategies for big data application based on their resource endowments and industry characteristics, ensuring that investments in data elements translate into tangible benefits. Finally, it is important to note that big data application relies on various complementary resource inputs. While adopting big data technologies, enterprises should also focus on enhancing their capacity to allocate and integrate supporting resources.

## Statement on AI-assistive tools

In the indicator construction section, this paper employs the Qwen-Turbo model provided by Alibaba Cloud to analyze annual reports of listed companies. It identifies sentences containing keywords related to big data applications and further determines whether the company has genuinely implemented such applications. Compared to traditional methods that rely solely on keyword extraction from annual reports, the approach based on large language models (LLMs) enables more accurate identification of authentic big data adoption behaviors. In recent years, numerous studies have adopted similar methodologies, utilizing LLMs to process unstructured textual data. For instance, Li et al.^30^ used an LLM to measure corporate culture and its economic consequences based on annual reports of listed companies, while Fang et al.^31^ extracted multi-dimensional unstructured information—such as policy objectives, target industries, policy instruments, and implementation mechanisms—from approximately three million Chinese industrial policy documents. This paper innovatively applies LLMs to conduct in-depth semantic mining of corporate annual reports, aiming to establish a more objective and accurate measurement system for corporate big data application.

## Supplementary Information

Supplementary Information.

## Data Availability

The datasets generated and analysed during the current study are available from the corresponding author on reasonable request.
