# Supplementary material for: Big data application and firm markups: evidence from China
Source: Sci Rep. 2026 Apr 8;16:11670. doi: 10.1038/s41598-026-43480-1 (PMC13061941; doi:10.1038/s41598-026-43480-1)
Supplement: Supplementary file 1 — Supplementary Information. [file 41598_2026_43480_MOESM1_ESM.docx]

# Online Appendix to "Big Data Application and Firm Markups: Evidence from China"

# Appendix 1:Supplementary Model Derivations

(1) Big Data Application and Firm Price Markup

The firm’s marginal cost is $MC=c+\frac{\delta}{f(a)}z$. Then the firm’s price markup is:

$$\begin{aligned} \mu(c)=p(c)-MC=\frac{1}{2}\left[ 1+\left( \beta-\frac{\delta}{f(a)} \right)\lambda\right](c_{D}-c)\# \end{aligned}$$

Let $x=\beta-\frac{\delta}{f(a)}$ , then $\lambda=\frac{\mathrm{Lx}}{4\theta\gamma-Lx^{2}}$ . The price markup can be expressed as $\mu(c)=\frac{1}{2}(1+x\lambda)(c_{D}-c)$. To examine the impact of big data application on price markup, we conduct derivative analysis on key variables step by step.

① First, consider the derivative of x with respect to the level of big data application a: $\frac{\partial x}{\partial a}=\frac{\partial}{\partial a}\left( \beta-\frac{\delta}{f(a)} \right)=\frac{\delta f^{'}(a)}{f(a)^{2}}$. Given the assumption that $f^{'}(a)>0$ and $f(a)>0$ , we have $\frac{\partial x}{\partial a}>0$.

② Second, analyze the derivative of $\lambda$ with respect to $a$. Since $\frac{\partial\lambda}{\partial x}=\frac{L(4\theta\gamma-Lx^{2})-Lx(-2Lx)}{(4\theta\gamma-Lx^{2})^{2}}=\frac{4L\theta\gamma+L^{2}x^{2}}{(4\theta\gamma-Lx^{2})^{2}}$, and $L>0$, $\theta>0$, $\gamma>0$, it follows that $\frac{\partial\lambda}{\partial x}>0$. Also, since$\frac{\partial x}{\partial a}>0$ , we have $\frac{\partial\lambda}{\partial a}=\frac{\partial\lambda}{\partial x}\cdot\frac{\partial x}{\partial a}>0$.

③ Take the derivative of price markup $\text{μ(c)}$ with respect to $\text{a}$: $\frac{\partial\mu(c)}{\partial a}=\frac{1}{2}(c_{D}-c)\left[ \lambda\frac{\partial x}{\partial a}+x\frac{\partial\lambda}{\partial a} \right]$ . Under reasonable economic assumptions (i.e., $c_{D}>c$, reflecting that the firm still has a production cost advantage), combined with $\frac{\partial x}{\partial a}>0$ , $\frac{\partial\lambda}{\partial a}>0$ , $\lambda>0$ , and $x=\beta-\frac{\delta}{f(a)}>0$ , it follows that $\frac{\partial\mu(c)}{\partial a}>0$ .

(2) Big Data Application, Product Innovation, and Firm Price Markup

By taking the derivative of product innovation with respect to big data application, we obtain:

$$\begin{aligned} \frac{\partial z*}{\partial a}=\frac{L\delta(c_{D}-c)(4\theta\gamma+Lx^{2})f^{'}(a)}{(4\theta\gamma-Lx^{2})^{2}f(a)^{2}}\# \end{aligned}$$

Since in the numerator: $L>0，\delta>0，c_{D}-c>0，4\theta\gamma+Lx^{2}>0$, and $f^{'}(a)>0$ ; and in the denominator: $(4\theta\gamma-Lx^{2})^{2}f(a)^{2}>0$ , it can be deduced that $\frac{\partial z_{i}^{*}}{\partial a}>0$.

# Appendix 2:Derivation of Firm Price Markup

Markup, defined as the ratio of price to marginal cost. This paper adopts the method of Loecker and Warzynski (2012) to calculate firms' markups. Assume the firm's production function is:

$$Y_{it}=F_{i}(L_{it},K_{it},M_{it},\omega_{it})$$

where $i$ denotes the firm, $t$ denotes the time period, $Y$ is the firm's output, $L$ is labor input, $K$is capital input, $M$ is intermediate input, and $\omega$ represents the firm's productivity. Consider the firm's cost minimization problem:

$$\min_{\{L_{it},K_{it},M_{it}\}} w_{it}L_{it}+r_{it}K_{it}+P_{it}^{M}M_{it}$$

$$\quad s.t.\quad F_{i}(L_{it},K_{it},M_{it},\omega_{it})=Y_{it}$$

where $w$ denotes the price of labor input,$r$ denotes the price of capital input, and $P^{M}$denotes the price of intermediate input. From the first-order condition, we obtain:

$$\frac{\partial F_{i}}{\partial M_{it}}\frac{M_{it}}{Y_{it}}=\frac{P_{it}}{MC_{it}}\frac{P_{it}^{M}M_{it}}{P_{it}Y_{it}}$$

where $P$ is the price level of the firm's final product, and$MC$ is the marginal cost. Define:

$$\frac{P_{it}^{M}M_{it}}{P_{it}Y_{it}}\equiv\alpha_{it}^{M}$$

$\alpha$ represents the share of intermediate input in the firm's total output, which can be directly calculated from the data;

$$\frac{\partial F_{i}}{\partial M_{it}}\frac{M_{it}}{Y_{it}}\equiv\theta_{it}^{M}$$

$\theta$ represents the elasticity of firm output with respect to intermediate input, which can be calculated by estimating production function parameters. This paper assumes a translog form production function and follows the approach of Ackerberg et al. (2015) to estimate production function parameters. The expression for the firm's markup rate is:

$${Markup}_{it}=\frac{\theta_{it}^{M}}{\alpha_{it}^{M}}$$

In the actual calculation process, labor input is measured as the natural logarithm of the number of employees, capital input is measured as the natural logarithm of net fixed assets, and firm output is measured as the natural logarithm of operating revenue. Intermediate input is calculated using the following accounting identity: Total intermediate input = Financial expenses + Management expenses + Sales expenses - Cash paid to and on behalf of employees - Depreciation and amortization + Cost of goods sold.

# Appendix 3: Indicator Validity Test

(1) Model Performance Comparison

Through a rigorous model training and validation process, we developed a high-performance text recognition model. This study compares the predictive performance of a large language model (LLM) with seven other text classification methods based on two hundred test text cases. The performance differences among the models are illustrated in Figure A1. Overall, the LLM achieved the best results across all evaluation metrics, particularly excelling in Macro-F1 and Micro-F1 scores, demonstrating its superior capability in handling complex texts and deep contextual information. In conclusion, owing to its powerful language understanding and generation abilities, the LLM significantly outperformed other traditional methods and feature-based models in this text classification task.


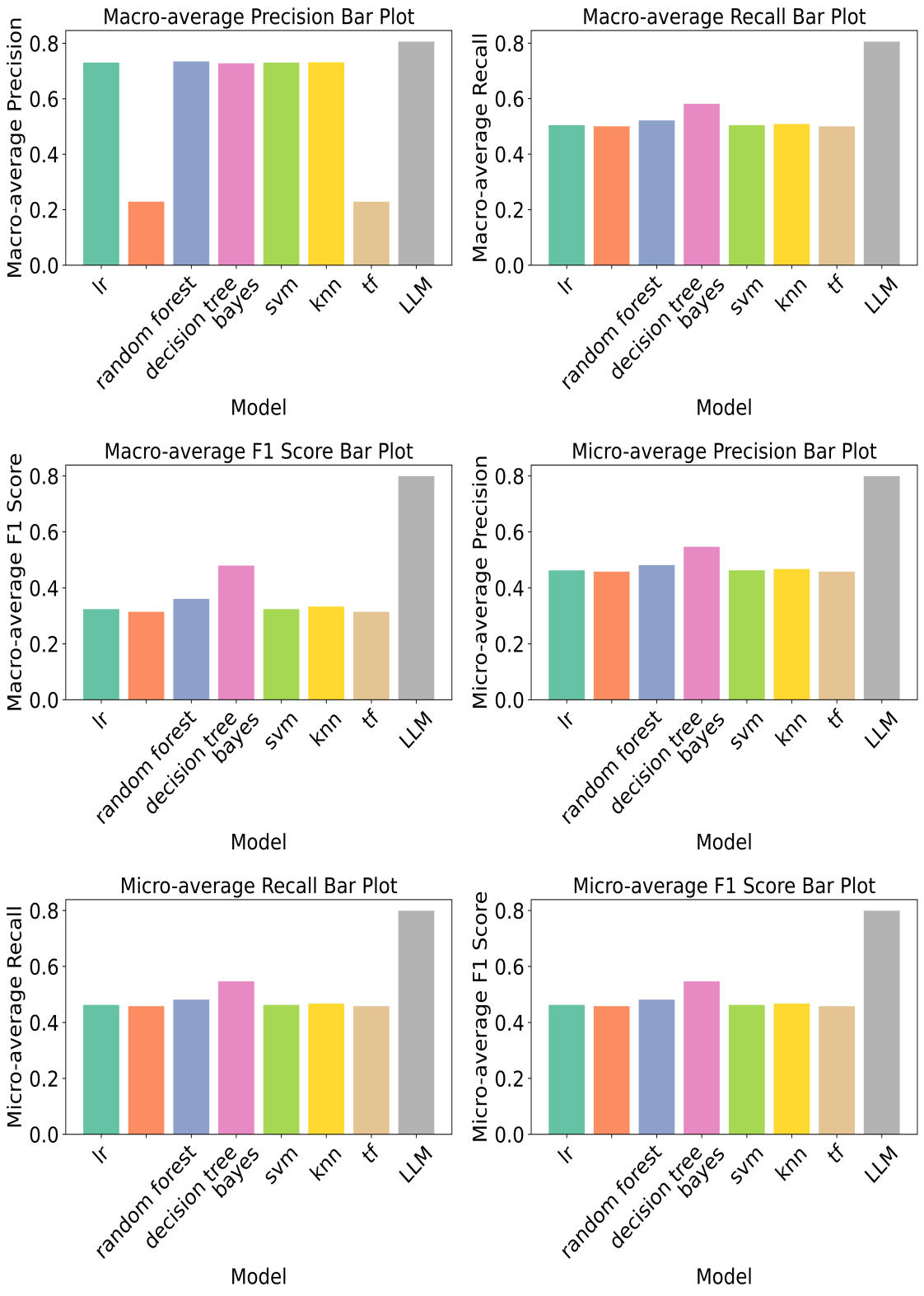


**Figure A1. Model Performance Comparison**

(2) Indicator Comparison

To validate the effectiveness of the big data application indicator constructed in this paper, regression analyses were conducted between this indicator and related metrics such as the scale of data-related intangible assets, the number of data analysis job postings, and the volume of digital technology patent applications. The study was carried out while controlling for the influence of other variables. The specific construction methods of the related indicators are detailed in the robustness test section of the main text, and the model specification remains consistent with the baseline regression. The results in Table A1 show a significant positive relationship between the big data indicator and each of the aforementioned metrics, further confirming that this indicator effectively reflects the close connection with the level of data-related intangible assets, the market demand for data analysis talent, and the activity level of digital technology innovation.

**Table A1. Indicator Validity Test**

|  | InBigdata | InBigdata | InBigdata |
| --- | --- | --- | --- |
| DigIntangibleAssets | 0.0829*** |  |  |
|  | (0.0134) |  |  |
| BigDataTalentRecruitment |  | 0.0419*** |  |
|  |  | (0.0059) |  |
| Digital Patents |  |  | 0.0372*** |
|  |  |  | (0.0106) |
| Controls | YES | YES | YES |
| Firm FE | YES | YES | YES |
| Year FE | YES | YES | YES |
| Number | 19,093 | 17,035 | 13,032 |
| R-squared | 0.8002 | 0.8368 | 0.8215 |

(3) Validation with Real-World Context

Table A2 reports the frequency counts of big data application keywords across various industries. The results reveal significant disparities in the adoption of big data technologies among different sectors in China. Digital-intensive industries—such as software and information technology services, computer and electronic equipment manufacturing, and internet-related services—exhibit the most extensive application of big data. In contrast, traditional sectors like agriculture, forestry, animal husbandry, fishing, mining, and textiles and apparel show relatively lower levels of adoption. This distribution pattern aligns closely with the 2021 China Academy of Information and Communications Technology estimates regarding the penetration rate of the digital economy across the three major industries in 2020, further corroborating the reliability of the measurement results presented here.

**Table A2. Frequency Counts of Big Data Application Keywords by Industry**

| Industry Name | Number | Industry Name | Number |
| --- | --- | --- | --- |
| Software and Information Technology Services | 55743 | Textile Industry | 529 |
| Manufacture of Computers, Communication, and Other Electronic Equipment | 16511 | Agricultural and Sideline Food Processing Industry | 524 |
| Manufacture of Electrical Machinery and Equipment | 7690 | Postal Service | 524 |
| Internet and Related Services | 7076 | Metal Products Industry | 513 |
| Manufacture of Special Purpose Equipment | 5857 | Broadcasting, Television, Film, and Audiovisual Production | 512 |
| Manufacture of Instruments and Apparatus | 2846 | Food Manufacturing Industry | 511 |
| Professional Technical Services | 2551 | Health Sector | 505 |
| Telecommunications, Radio and Television, and Satellite Transmission Services | 2471 | Public Facilities Management | 477 |
| Manufacture of General Purpose Equipment | 2405 | Rubber and Plastics Products Industry | 452 |
| Retail Trade | 2360 | Furniture Manufacturing Industry | 448 |
| Business Services | 2218 | Building Decoration and Other Construction | 418 |
| Wholesale Trade | 1973 | Smelting and Pressing of Ferrous Metals | 413 |
| Manufacture of Raw Chemical Materials and Chemical Products | 1693 | Manufacture of Cultural, Educational, Arts, Crafts, Sports, and Entertainment Products | 384 |
| Pharmaceutical Manufacturing | 1593 | Manufacture of Liquor, Beverages, and Refined Tea | 337 |
| Real Estate Industry | 1283 | Water Transport Industry | 329 |
| News and Publishing Industry | 897 | Smelting and Pressing of Non-Ferrous Metals | 313 |
| Manufacture of Textile Apparel and Apparel | 831 | Warehousing Industry | 286 |
| Automobile Manufacturing | 831 | Other Manufacturing Industries | 284 |
| Manufacture of Railway, Ship, Aerospace, and Other Transport Equipment | 802 | Manufacture of Leather, Fur, Feather, and Related Products and Footwear | 273 |
| Ecological Protection and Environmental Management | 750 | Supporting Activities for Mining | 236 |
| Civil Engineering Construction | 680 | Cultural and Arts Industry | 213 |
| Production and Supply of Electric Power and Heat Power | 663 | Chemical Fiber Manufacturing | 192 |
| Paper and Paper Products Industry | 633 | Research and Experimental Development | 179 |
| Road Transport Industry | 536 | Printing and Reproduction of Recording Media | 170 |
| Non-Metallic Mineral Products Industry | 534 | Air Transport Industry | 170 |

# Appendix 4: Additional Robustness Tests

(1) Estimation Results of Big Data Application Sub-Dimensions

In the baseline regression, this study employs a comprehensive indicator of enterprise big data application as the core explanatory variable. To further verify the reliability of the results, subsequent analyses constructed alternative indicators for robustness tests based on five sub-dimensions: conceptual layer, foundational layer, technological layer, organizational layer, and application layer. The results in Table A3 show that, except for the organizational layer and the application layer, the estimated coefficients of the core variables corresponding to the conceptual layer, foundational layer, and technological layer are all significantly positive at the 1% level, thereby confirming the robustness of the baseline findings across the primary dimensions.

The potential reasons for the non-significant results in the organizational and application layers include: first, the impact of organizational change exhibits a lag, requiring a certain period to translate into observable market performance (i.e., enterprise markup rate); second, the effectiveness of the application layer is characterized by heterogeneity and cost constraints, as outcomes vary significantly across different enterprises and scenarios, and the high upfront costs of trial-and-error and technology integration may temporarily diminish its overall positive effect on the markup rate.

**Table A3. Estimation Results of Big Data Application Sub-Dimensions**

|  | (1) | (2) | (3) | (4) | (5) |
| --- | --- | --- | --- | --- | --- |
|  | Markup | Markup | Markup | Markup | Markup |
| LnConcept | 0.0138*** |  |  |  |  |
|  | (0.0024) |  |  |  |  |
| LnBasic |  | 0.0118*** |  |  |  |
|  |  | (0.0026) |  |  |  |
| LnTechnical |  |  | 0.0211*** |  |  |
|  |  |  | (0.0041) |  |  |
| LnOrganizational |  |  |  | 0.0233 |  |
|  |  |  |  | (0.0152) |  |
| LnApplication |  |  |  |  | -0.0017 |
|  |  |  |  |  | (0.0048) |
| Controls | YES | YES | YES | YES | YES |
| Firm FE | YES | YES | YES | YES | YES |
| Year FE | YES | YES | YES | YES | YES |
| Observations | 19,555 | 19,555 | 19,555 | 19,555 | 19,555 |
| R-squared | 0.7977 | 0.7972 | 0.7973 | 0.7967 | 0.7966 |

(2) Adopting Measurement Methods from Classical Literature

To ensure the robustness of the baseline regression results, this study draws on well-established and widely adopted measurement methods in the academic field to conduct a replacement test for the core explanatory variable. Specifically, we employ two alternative indicators: First, following the approach of Zhang et al. (2021), we construct an indicator based on the frequency of keywords such as "big data," "massive data," and "data center" in corporate annual reports. Second, we adopt the measurement used by Bai et al. (2021), which is based on the frequency of terms such as "big data," "data mining," and "VR/AR/MR."

As shown in Table A4, the estimated coefficients for both the variable based on the Zhang et al. dictionary (LnBigdata_Zhang) and the variable based on the Bai et al. dictionary (LnBigdata_Bai) are significantly positive at the 1% level, with the coefficient magnitudes being very similar. This indicates that the core findings are not sensitive to the choice of different but mainstream keyword dictionaries. The regression results are highly consistent with the baseline conclusions, further confirming that big data has a significant positive effect on firms' price markup, thereby enhancing the robustness of the study's findings.

**Table A4. Estimation Results with Replaced Core Explanatory Variable**

|  | (1) | (2) |
| --- | --- | --- |
|  | Markup | Markup |
| LnBigdata_Zhang | 0.0111*** |  |
|  | (0.0026) |  |
| LnBigdata_Bai |  | 0.0108*** |
|  |  | (0.0025) |
| Controls | YES | YES |
| Firm FE | YES | YES |
| Year FE | YES | YES |
| Observations | 16,678 | 16,678 |
| R-squared | 0.7989 | 0.7989 |

Note: Zhang et al. (2021) constructed corresponding indicators by counting the frequency of keywords such as "big data, massive data, data center, information assets, datafication, computing power" in corporate annual reports. Bai et al. (2021), on the other hand, adopted a measurement index based on the frequency of terms such as "big data, data mining, text mining, data visualization, heterogeneous data, credit reporting, augmented reality (AR), mixed reality (MR), virtual reality (VR), data science."

(3) Other Robustness Test Methods

This study also conducted the following robustness tests: ① Incorporated control variables at the city and industry levels into the baseline model, including industry concentration (measured by the HHI index), regional economic development level, and government technology expenditure; ② Adjusted the clustered standard errors to both industry and city levels to enhance the reliability of statistical inference; ③ Applied winsorization to the top and bottom 1% of all continuous variables in the model to mitigate the potential impact of outliers on estimation results; ④ Added an IT industry dummy variable to control for industry heterogeneity; ⑤ Introduced a pandemic time dummy variable to control for the impact of the pandemic shock on the results; ⑥ In the regression model, we incorporate both city fixed effects and industry fixed effects to control for time-invariant regional and sectoral characteristics, respectively. Table A5 reports the estimation results, where the coefficient of LnBigdata remains significantly positive at the 1% level, consistent with the baseline conclusion, indicating strong robustness of the original estimation results. This further confirms that big data has a significant promoting effect on firms' price markups.

**Table A5. Robustness Tests**

|  | (1) | (2) | (3) | (4) | (5) | (6) |
| --- | --- | --- | --- | --- | --- | --- |
|  | Markup | Markup | Markup | Markup | Markup | Markup |
|  | City/industry controls | Cluster (Ind) | Cluster (City) | IT  Dummy | COVID Dummy | Controls for City & Ind FE |
| LnBigdata | 0.0116*** | 0.0125*** | 0.0125*** | 0.0127*** | 0.0125*** | 0.0136*** |
|  | (0.0024) | (0.0043) | (0.0023) | (0.0024) | (0.0024) | (0.0023) |
| Controls | YES | YES | YES | YES | YES | YES |
| Firm FE | YES | YES | YES | YES | YES | YES |
| Year FE | YES | YES | YES | YES | YES | YES |
| Observations | 17,431 | 19,555 | 19,555 | 19,555 | 19,555 | 19,554 |
| R-squared | 0.8082 | 0.7975 | 0.7975 | 0.7980 | 0.7975 | 0.8117 |

# Appendix 5: Mechanism Test

Theoretical analysis in this paper proposes that enterprises' application of big data can positively contribute to their markup by promoting product innovation and production efficiency. To examine whether this mediating mechanism holds, this study employs the classic mediating effect model (Baron & Kenny, 1986) and constructs the following three-stage regression models:

$$\text{Markup}_{\text{it}}\text{=}\text{α}_{\text{1}}\text{+}\text{β}_{\text{1}}\text{ }\text{LnBigData}_{\text{it}}\text{+}\text{γ}_{\text{1}}\text{ }\text{X}_{\text{it}}\text{+}\text{μ}_{\text{i}\text{1}}\text{+}\text{λ}_{\text{t}\text{1}}\text{+}\text{ϵ}_{\text{it}\text{1}}$$

$$\mathrm{Mediator}_{it}=\alpha_{2}+\beta_{2} \text{LnBigData}_{it}+\gamma_{2} \mathbf{X}_{it}+\mu_{i2}+\lambda_{t2}+\epsilon_{it2}$$

$$\mathrm{Markup}_{it}=\alpha_{3}+\beta_{3} \text{LnBigData}_{it}+\theta\mathrm{Mediator}_{it}+\gamma_{3} \mathbf{X}_{it}+\mu_{i3}+\lambda_{t3}+\epsilon_{it3}$$

This study selects product innovation and production efficiency as mediating variables and incorporates firm fixed effects (μi) and year fixed effects (λt) to control for unobservable firm heterogeneity and time trends. Product innovation is proxied by R&D intensity (i.e., the ratio of R&D expenditure to total assets in the current period, denoted as RDsz), an indicator that effectively reflects a firm's resource investment in exploratory innovation and product improvement. Production efficiency is measured using total factor productivity (TFP) estimated based on the ACF method (Ackerberg et al., 2015). This method controls for simultaneity bias and sample selection issues, enabling a more accurate reflection of a firm’s actual production efficiency. To ensure the reliability of the findings, this paper follows the mediation analysis steps proposed by Fan et al. (2025), employing Sobel tests and Bootstrap methods sequentially for statistical testing of the mediation pathways.

Table A6 presents the results of the mechanism test with product innovation as the mediating variable. Column (1) shows that the coefficient of the core explanatory variable LnBigdata is significantly positive at the 1% level, indicating that big data application significantly enhances firm markup rates, verifying its direct promotional effect.

Columns (2) and (3) report the impact of big data application on R&D intensity. Regardless of whether control variables are included, the coefficients of LnBigdata on the mediating variable RDsz are significantly positive, satisfying the initial condition for mediation effect testing—namely, that big data application can effectively increase firm R&D intensity. Columns (4) and (5) further introduce the mediating variable RDsz into the baseline model. The results show that the coefficients of RDsz are significantly positive at the 1% level, while the direct effect coefficients of LnBigdata, although slightly reduced compared to earlier estimates, remain significant at the 1% level. This preliminarily indicates that R&D intensity plays a partial mediating role in the process through which big data application influences firm markup rates.

To enhance the robustness of the above conclusions, Sobel tests and Bootstrap tests were conducted. The Sobel test Z-statistics corresponding to columns (4) and (5) are both significant, and the 95% confidence intervals constructed based on 1,000 Bootstrap resamples do not include zero, further supporting the significant mediating effect of R&D intensity. According to the model that includes all control variables, the mediating effect of R&D intensity accounts for approximately 6.69% of the total effect.

**Table A6. Mechanism Test: Product Innovation**

|  | (1) | (2) | (3) | (4) | (5) |
| --- | --- | --- | --- | --- | --- |
|  | Markup | RDsz | RDsz | Markup | Markup |
| LnBigdata | 0.0125*** | 0.0010*** | 0.0010*** | 0.0084*** | 0.0080*** |
|  | (0.0024) | (0.0002) | (0.0002) | (0.0023) | (0.0022) |
| RDsz |  |  |  | 0.7370*** | 0.6913*** |
|  |  |  |  | (0.1518) | (0.1513) |
| Controls | YES | NO | YES | NO | YES |
| FE | YES | YES | YES | YES | YES |
| Observations | 19,555 | 21,769 | 21,748 | 19,144 | 19,135 |
| R-squared | 0.7975 | 0.8896 | 0.8900 | 0.8031 | 0.8059 |
| Sobel Z value  (p-value) |  |  |  | 2.7770***  (0.0055) | 2.7670***  (0.0056) |
| Bootstrap (1000 times) 95% conf. Interval |  |  |  | [0.0003,0.0009] | [0.0002,0.0009] |
| Proportion of total effect that is mediated |  |  |  | 0.0669 | 0.0669 |

Note: The Sobel Z-value (with p-value in parentheses) is a traditional statistic used to test the significance of the mediating effect. If the p-value is less than the significance level (e.g., 0.05), it indicates the presence of a mediating effect. The Bootstrap 95% confidence interval is based on 1,000 resampling repetitions. If the interval does not contain 0, it likewise supports the conclusion that the mediating effect is significant. This method is more robust than the Sobel test. The proportion of the mediating effect refers to the share of the mediating effect in the total effect, which is used to measure the explanatory power of the mediating variable. The same applies below.

Table A7 presents the results of the mechanism test with total factor productivity (TFP) as the mediating variable. To examine the mechanism of TFP, Models (2) and (3) test the impact of LnBigdata on the mediating variable TFP. The results show that, regardless of whether control variables are included, the coefficient of LnBigdata on TFP is significantly positive, satisfying the first condition for the mediating effect. This indicates that the application of big data can significantly enhance the firm’s production efficiency. Models (4) and (5) incorporate the mediating variable TFP on the basis of the benchmark regression, and it is found that the coefficients of TFP are significantly positive at the 1% level. At the same time, the coefficient of LnBigdata decreases, suggesting that production efficiency plays a partial mediating role.

To ensure the robustness of the conclusion, we further report the results of the Sobel test and the Bootstrap test. The Sobel Z statistics corresponding to Models (4) and (5) are 5.351 and 4.281, respectively, both significant at the 1% level. The 95% confidence intervals constructed using the Bootstrap method do not include zero, jointly verifying the robustness of the mediating effect. According to Model (5), which includes all control variables, the mediating effect transmitted through production efficiency accounts for approximately 49.77% of the total effect.

**Table A7. Mechanism Test: Production Efficiency**

|  | (1) | (2) | (3) | (4) | (5) |
| --- | --- | --- | --- | --- | --- |
|  | Markup | TFP | TFP | Markup | Markup |
| LnBigdata | 0.0125*** |  |  |  |  |
|  | (0.0024) |  |  |  |  |
| TFP |  |  |  |  |  |
|  |  |  |  |  |  |
| Controls | YES | NO | YES | NO | YES |
| FE | YES | YES | YES | YES | YES |
| Observations |  |  |  |  |  |
| R-squared |  |  |  |  |  |
| Sobel Z value  (p-value) |  |  |  | 5.351***  (0.0000) | 4.281***  (0.0000) |
| Bootstrap (1000 times) 95% conf. Interval |  |  |  | [0.0071,0.0096] | [0.0056,0.0085] |
| Proportion of total effect that is mediated |  |  |  | 0.6354 | 0.4977 |

# References

1. Ackerberg, D. A., Caves, K., & Frazer, G. (2015). Identification properties of recent production function estimators. Econometrica, 83(6), 2411-2451. <https://doi.org/10.3982/ECTA13408>
2. Baron, R. M., & Kenny, D. A. (1986). The moderator–mediator variable distinction in social psychological research: Conceptual, strategic, and statistical considerations. Journal of personality and social psychology, 51(6), 1173. https://doi.org/10.1037/0022-3514.51.6.1173
3. Fan, W., Xu, H., Cheng, S., & Yang, F. (2025). How do health shocks affect household energy poverty?. Energy Economics, 108884.https://doi.org/10.1016/j.eneco.2025.108884
4. Loecker, J. D., & Warzynski, F. (2012). Markups and firm-level export status. American economic review, 102(6), 2437-2471.https://doi.org/10.1257/aer.102.6.2437
